# Electronic properties of the coronene series from thermally-assisted-occupation density functional theory†

**DOI:** 10.1039/c8ra01336e

**Published:** 2018-10-08

**Authors:** Chia-Nan Yeh, Can Wu, Haibin Su, Jeng-Da Chai

**Affiliations:** Department of Physics, National Taiwan University Taipei 10617 Taiwan jdchai@phys.ntu.edu.tw; School of Materials Science and Engineering, Nanyang Technological University 50 Nanyang Avenue Singapore 639798 Republic of Singapore haibinsu@ust.hk; Department of Chemistry, The Hong Kong University of Science and Technology Hong Kong China haibinsu@ust.hk; Center for Theoretical Physics, National Taiwan University Taipei 10617 Taiwan; Center for Quantum Science and Engineering, National Taiwan University Taipei 10617 Taiwan

## Abstract

To fully utilize the great potential of graphene in electronics, a comprehensive understanding of the electronic properties of finite-size graphene flakes is essential. While the coronene series with *n* fused benzene rings at each side (designated as *n*-coronenes) are possible structures for opening a band gap in graphene, their electronic properties are not yet fully understood. Nevertheless, because of their radical character, it remains very difficult to reliably predict the electronic properties of the larger *n*-coronenes with conventional computational approaches. In order to circumvent this, the various electronic properties of *n*-coronenes (*n* = 2–11) are investigated using thermally-assisted-occupation density functional theory (TAO-DFT) [J.-D. Chai, *J. Chem. Phys.*, 2012, **136**, 154104], a very efficient electronic structure method for studying nanoscale systems with strong static correlation effects. The ground states of the larger *n*-coronenes are shown to be polyradical singlets, where the active orbitals are mainly localized at the zigzag edges.

## Abstract 2

To fully utilize the great potential of graphene in electronics, a comprehensive understanding of the electronic properties of finite-size graphene flakes is essential.

## Graphical abstract

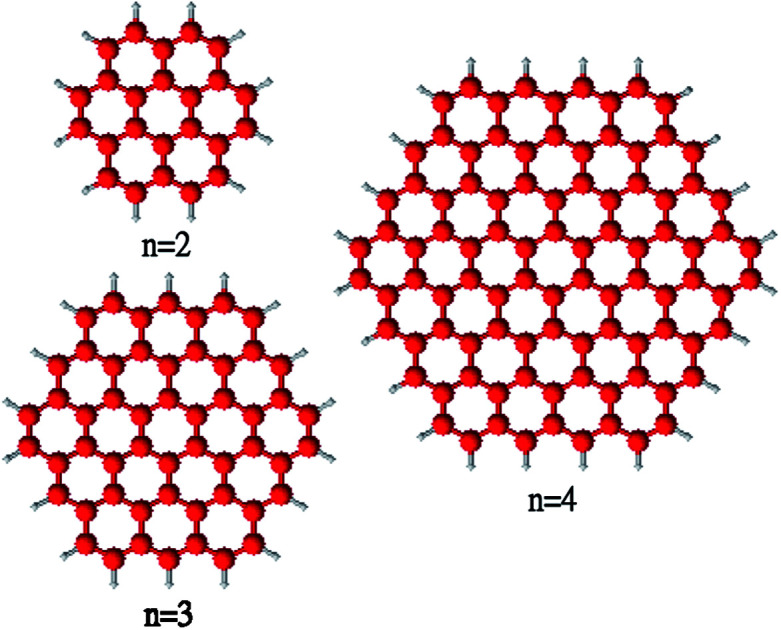

## I. Introduction

The discovery of graphene has attracted much scientific attention in recent years, owing to its fascinating properties and promising applications in industry.^1–5^ For example, its high carrier mobility, saturation velocity, and long spin diffusion length have enabled us to build promising graphene-based electronics and spintronics.^1,2,6^ Nevertheless, its applications have been partially impeded by its zero band gap. One scenario to introduce a band gap in graphene is the finite-size effect by cutting infinite graphene lattice into specific geometrical arrangements with smooth edges attached by hydrogen atoms.^7–9^ The resulting planar hydrocarbons can be classified into different categories according to the shape, geometry, size, or edges.^10,11^ Besides, all these fragments with a size less than 30 nm are referred to as graphene quantum dots (GQDs).^12,13^

There are two main approaches for the preparation of GQDs: the top-down^14^ and bottom-up^15^ approaches. The top-down approach is to prepare the wanted samples through cutting graphene sheets in nanoscale technology. This approach demands for expensive equipment, and is hard to control precisely on the zigzag or armchair edges. By contrast, the bottom-up approach is to obtain the designed samples through oxidation, cage opening, and fragmentation of fullerene (C_60_) or other carbon fibers with chemical breakdown. Some studies have reported the successful synthesis of GQDs within a very wide range of diameters as well as the corresponding visible photoluminescence (PL) spectra.^10,16–18^ Kim *et al.* have fabricated a series of GQDs with an average diameter ranging from 5 to 35 nm through chemical cutting of graphene sheets.^18^ Their results have shown that for the GQDs keeping circular shape less than 17 nm, the peak frequency of G band is increasing *versus* increasing size. While for those larger than 17 nm, the value is decreasing *versus* increasing size. Pan *et al.* have reported a chemical route to obtain ultrafine GQDs from graphene sheets.^16^ They observed a strong blue PL peak and two characteristic PL excitation (PLE) peaks. The novel optical phenomena were explained by the carbene-like triplet ground state, which originates from the free zigzag edge sites. All these findings, together with other studies,^19–24^ have proven that the electronic and optical properties of 2-dimensional (2D) hydrocarbons cutting from infinite graphene sheets are highly affected by their nanostructure, size, shape, and periphery conditions, which can be qualitatively described in terms of the superposed Clar's structures or superaromatic stabilization energy.^25–29^

Among several 2D structures of hydrocarbons, quasi-one-dimensional strips are the most popular ones. They are officially referred to as graphene nanoribbons (GNRs), which have been actively studied in recent years.^7–9,19–24,30–42^ In 2004, Bendikov *et al.* adopted the complete-active-space self-consistent-field (CASSCF) method to study acenes (*i.e.*, the narrowest zigzag GNRs),^31^ showing that the larger acenes (*e.g.*, those larger than pentacene) possess radical character in their ground states. Similar results were also found from other high-level *ab initio* calculations.^32,36^

**Fig. 1:**
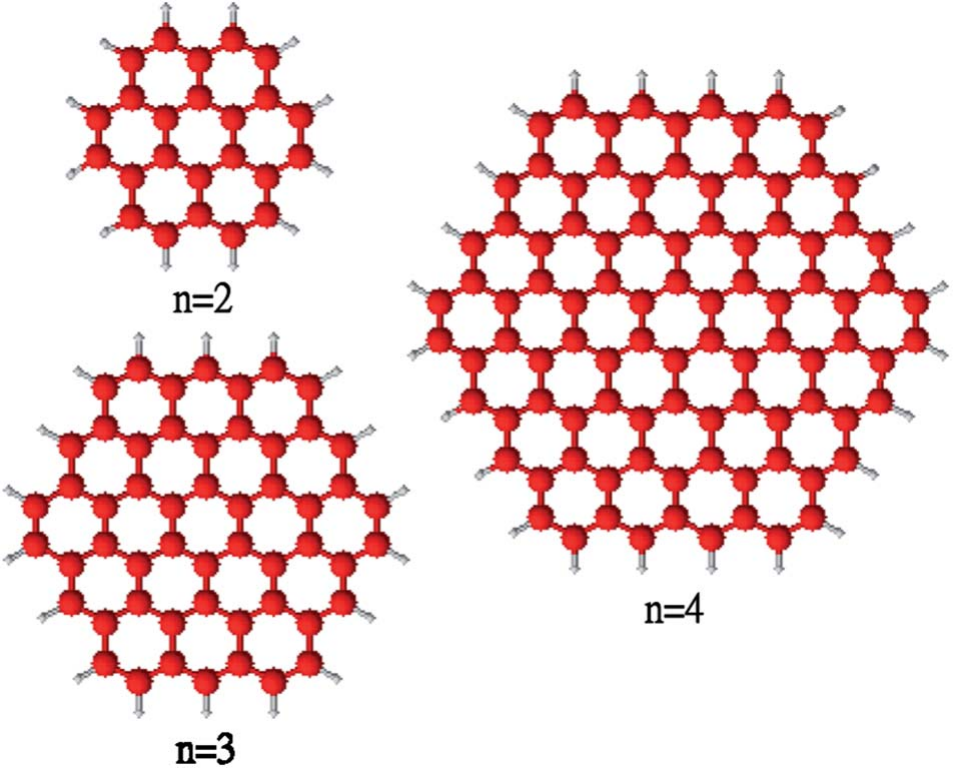
Structure of *n*-coronene, containing *n* fused benzene rings at each side.

Despite the intensive studies of strip-like-shape hydrocarbons, the understanding of the correlations between the electronic properties and geometrical arrangements of 2D hydrocarbons remains rather limited. Among other geometrical arrangements, a class of 2D hydrocarbons that deserve our exploration are the regular-hexagon-shaped benzenoid hydrocarbons, which can be extended from coronene (also called superbenzene, composed of six peri-fused benzene rings). The *D*_6h_-symmetric hexagon-shaped series with chemical stoichiometry C_6*n*^2^_H_6*n*_ are referred to as coronene (*n* = 2), circumcoronene (*n* = 3), circumcircumcoronene (*n* = 4), and so on, with *n* being the number of fused benzene rings at each side. For brevity, C_6*n*^2^_H_6*n*_ is designated as *n*-coronene in this work (see Fig. 1). In 2009, Ikäläinen *et al.* performed a computational study on *n*-coronenes (up to *n* = 4),^43^ demonstrating that the energy difference between the highest occupied molecular orbital (HOMO) and lowest unoccupied molecular orbital (LUMO), *i.e.*, the HOMO–LUMO gap, of *n*-coronene decreases very slowly with the length scale (*n*). This suggests that the smaller *n*-coronenes should possess non-radical character in their ground states. However, as *n*-coronene (*n* → ∞) reduces to graphene, the larger *n*-coronenes are expected to have vanishingly small HOMO–LUMO gaps, and hence possess radical character in their ground states. Accordingly, with increasing *n*, there should be a transition from the non-radical character of the smaller *n*-coronenes to the radical character of the larger *n*-coronenes. However, the existing studies of the larger *n*-coronenes (*e.g.*, *n* ≥ 5) remain too limited to address this issue.^44^ Besides, it remains unclear if the larger *n*-coronenes possess di-, tri-, or even higher polyradical ground states.

On the experimental side, there were scarce experimental studies of the members larger than coronene. Although the smaller *n*-coronenes are expected to be energetically stable, it remains challenging to realize the precise regular-hexagon-shaped benzenoid hydrocarbons with smooth edges. This may be due to the following four reasons: (1) in order to cut graphene sheets or open the cage fullerene to make graphene flakes, the process involves a series of chemical reactions with many byproducts generated, where the fraction of desired organic structures are very low; (2) it is very hard to separate the pure molecules due to similar chemical properties; (3) due to a series of redox reactions, the prepared samples are usually attached by derivatives rather than pure hydrocarbons; (4) the expensive equipment, long manufacture period, raw material toxicity procedures, and some other realistic factors also constrain the experimental results.^16,45–50^ Therefore, due to these technical reasons, the fabrication of circumcoronene and the larger members remains very challenging.

On the theoretical side, Kohn–Sham density functional theory (KS-DFT)^51^ with the widely used exchange-correlation (XC) density functionals is computationally efficient, but can yield enormous errors for systems with strong static correlation effects (*i.e.*, systems possessing strong multi-reference or radical character).^52^ To reliably describe the properties of systems with radical character, accurate *ab initio* multi-reference methods are usually needed.^53^ Nonetheless, accurate multi-reference calculations are prohibitively expensive for large systems (especially for geometry optimization). Consequently, it remains very challenging to investigate the electronic properties of the larger *n*-coronenes using conventional electronic structure methods.

To address these challenges with minimum computational complexity, in 2012, Chai developed thermally-assisted-occupation density functional theory (TAO-DFT)^35^ for studying large ground-state systems with strong static correlation effects. TAO-DFT has similar computational cost as KS-DFT for single-point energy and analytical nuclear gradient calculations, and reduces to KS-DFT in the absence of strong static correlation. In contrast to KS-DFT, TAO-DFT is a density functional theory with fractional orbital occupations, wherein strong static correlation is explicitly described by the entropy contribution (see eqn (26) of ref. 35), a function of the fictitious temperature and orbital occupation numbers. Note that the entropy contribution is completely missing in KS-DFT. Unlike highly accurate *ab initio* multi-reference methods, TAO-DFT is computationally efficient, and hence promising for the study of large polyradical systems. With some trivial modifications, existing semilocal and hybrid XC density functionals in KS-DFT may also be adopted in TAO-DFT.^38,42^ Recently, a self-consistent scheme for the determination of the fictitious temperature in TAO-DFT has been developed,^54^ further extending the applicability of TAO-DFT for a very wide range of systems. Since 2015, TAO-DFT has been widely applied to study several nanoscale systems with strong static correlation effects, including zigzag GNRs,^35,38,39,41,42^ cyclacenes,^55^ alternant polycyclic aromatic hydrocarbons (PAHs),^29^ and linear carbon chains.^56^ The results obtained with TAO-DFT have been shown to be in good agreement with the available experimental and highly accurate *ab initio* data. Besides, recent studies^29,40^ have shown that the orbital occupation numbers obtained with TAO-DFT are qualitatively similar to the natural orbital occupation numbers (NOONs), *i.e.*, the eigenvalues of one-electron reduced density matrix,^57^ obtained with highly accurate *ab initio* multi-reference methods, which can be very useful for the assessment of the possible polyradical character of large systems.

In view of its reasonable accuracy and computational efficiency for nanoscale systems with strong static correlation effects, in this work, TAO-DFT is employed to study the various electronic properties of *n*-coronenes (*n* = 2–11). To make our data convincing, our results are also compared with the available experimental data as well as those obtained with various XC density functionals in KS-DFT. However, experimental and high-level *ab initio* data for *n*-coronenes (*n* = 3–11) are currently unavailable for comparison.

### II. Computational details

All calculations are performed with a development version of Q-Chem 4.0,^58^ using the 6-31G basis set with the numerical grid containing 75 Euler-Maclaurin radial grid points and 302 Lebedev angular grid points. Results are computed using TAO-LDA,^35^*i.e.*, TAO-DFT with the local density approximation (LDA) XC density functional^59,60^ and the LDA *θ*-dependent density functional *E*^LDA^_*θ*_ (see eqn (41) of ref. 35) with the fictitious temperature *θ* = 7 mhartree (see ref. 35).

On the basis of the physical arguments given in Section III(E) of ref. 35 and the numerical investigations presented in Section IV of ref. 35, the static correlation energy of a system can be properly described by the entropy contribution [*i.e.*, a function of the fictitious temperature and orbital occupation numbers (an implicit density functional)], even when a local XC density functional is employed in TAO-DFT. Similar to the static correlation energy of a system, the entropy contribution in TAO-DFT is always nonpositive, yielding insignificant contributions for a single-reference system, and significantly lowering the total energy of a multi-reference system.

For comparison, some results are also computed using KS-LDA and KS-B3LYP (*i.e.*, KS-DFT with the LDA^59,60^ and B3LYP^61^ XC density functionals, respectively). Since TAO-LDA with *θ* = 0 reduces to KS-LDA, we examine the performance of KS-LDA here to evaluate the importance of TAO-LDA.

To determine the ground state of *n*-coronene (*n* = 2–11), we perform spin-unrestricted TAO-LDA, KS-LDA, and KS-B3LYP calculations for the lowest singlet and triplet energies of *n*-coronene on the respective geometries that were optimized at the same level of theory. The singlet–triplet energy (ST) gap of *n*-coronene is calculated as (*E*_T_ − *E*_S_), the energy difference between the lowest triplet (T) and singlet (S) states of *n*-coronene. When performing spin-unrestricted calculations on systems with an even number of electrons, it is usually necessary to break alpha/beta symmetry in the initial guess to ensure that the lowest energy solution is obtained. In our spin-unrestricted calculations, we have considered explicit symmetry breaking of the initial density guess (*i.e.*, *not* a symmetric closed-shell initial guess). Specifically, 30% of LUMO is added to HOMO for the alpha orbitals (but not for the beta orbitals) to break alpha/beta symmetry in the initial guess. In addition, our preliminary examinations show that the lowest singlet energies of *n*-coronenes (*n* = 2–8) [*i.e.*, the smaller *n*-coronenes (*e.g.*, *n* < 5) and some larger *n*-coronenes (*e.g.*, *n* = 5–8)] obtained from spin-unrestricted calculations with different initial guesses (*e.g.*, given by swapping the alpha frontier orbitals (*i.e.*, HOMO−1, HOMO, LUMO, and LUMO+1), but not the beta orbitals) are essentially the same (*i.e.*, within the numerical accuracy of our calculations) as those reported in this work. Our preliminary TAO-LDA results show that the 6-31G basis set adopted in this work should be reasonably large, especially for the larger *n*-coronenes (see Table S1 in ESI†).

In addition, we adopt the expectation value of the total spin-squared operator, 〈*Ŝ*^2^〉, to measure the degree of spin contamination in KS-DFT. Note that for a system with strong multi-reference character (*i.e.*, radical character), the value of 〈*Ŝ*^2^〉 obtained with conventional XC density functionals in KS-DFT can be very different (*e.g.*, more than 10% difference)^62^ from the exact value S (S + 1), where S can be 0 (singlet), 1/2 (doublet), 1 (triplet), 3/2 (quartet), and so on. For such a system, conventional XC density functionals in KS-DFT can yield inaccurate results. To adequately predict the electronic properties of systems with radical character, it can be essential to employ accurate *ab initio* multi-reference methods^53^ for small-sized systems or TAO-DFT^35,38,42^ for medium- to large-sized systems.

## III. Results and discussion

### A. Singlet–triplet energy gap

**Fig. 2:**
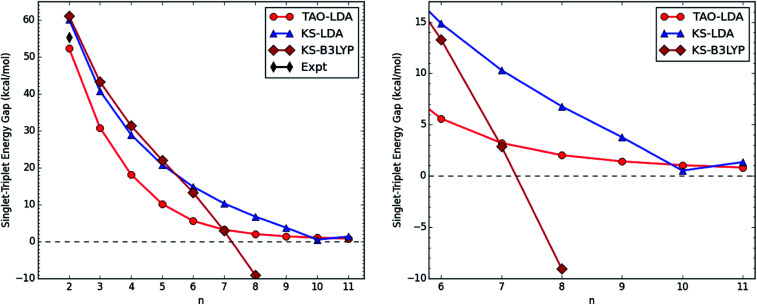
Singlet–triplet energy gap of *n*-coronene (left: *n* = 2–11; right: *n* = 6–11), obtained with spin-unrestricted TAO-LDA, KS-LDA, and KS-B3LYP. Here, the experimental data is taken from the literature.^50^

Fig. 2 shows the ST gap (*E*_ST_) of *n*-coronene, obtained with spin-unrestricted TAO-LDA, KS-LDA, and KS-B3LYP. For *n* = 2, the calculated ST gaps are in good agreement with the available experimental data.^50^ However, in contrast to the smooth *E*_ST_ curve obtained with TAO-LDA, the *E*_ST_ curves obtained with KS-LDA and KS-B3LYP are unsmooth for *n* ≥ 9 and for *n* ≥ 6, respectively. For KS-B3LYP, *n*-coronene (*n* = 8) is even predicted to have a triplet ground state, which is in strong contrast to the results obtained with TAO-LDA and KS-LDA (see Table S2 in ESI†).

To investigate the causes of discrepancies, the values of 〈*Ŝ*^2^〉 for the lowest singlet (see Table 1) and triplet (see Table 2) states of *n*-coronene are obtained with spin-unrestricted KS-LDA and KS-B3LYP. Based on the calculated values of 〈*Ŝ*^2^〉, the lowest singlet and triplet states of the smaller *n*-coronene possess single-reference (non-radical) character (*i.e.*, with no spin contamination or the calculated 〈*Ŝ*^2^〉 is very close to S (S + 1)). However, with the increase of the side length, the lowest singlet and triplet states of *n*-coronene are increasingly affected by spin contamination (*i.e.*, the artificial mixing of different electronic spin-states), implying that the larger *n*-coronene should possess increasing multi-reference (radical) character in the lowest singlet and triplet states. With KS-LDA, the effect of spin contamination becomes noticeable for *n* ≥ 9, while with KS-B3LYP, the effect of spin contamination becomes noticeable for *n* ≥ 6. Besides, the effect of spin contamination for the lowest triplet state of *n*-coronene is found to be more severe than that for the lowest singlet state of *n*-coronene. As spin contamination is not a systematic error, the difference in energy between states can be adversely affected. Therefore, for the larger *n*-coronenes, the unsmooth *E*_ST_ curves obtained with KS-LDA and KS-B3LYP should be artifacts closely related to spin contamination.

**Table 1:** Expectation value of the total spin-squared operator 〈*Ŝ*^2^〉 for the lowest singlet state of *n*-coronene, obtained with spin-unrestricted KS-LDA and KS-B3LYP

*n*	2	3	4	5	6	7	8	9	10	11
KS-LDA	0.0000	0.0000	0.0000	0.0000	0.0000	0.0000	0.0003	0.0124	0.0075	2.8196
KS-B3LYP	0.0000	0.0002	0.0002	0.0014	0.0087	0.0009	0.0095	—	—	—

**Table 2:** Expectation value of the total spin-squared operator 〈*Ŝ*^2^〉 for the lowest triplet state of *n*-coronene, obtained with spin-unrestricted KS-LDA and KS-B3LYP

*n*	2	3	4	5	6	7	8	9	10	11
KS-LDA	2.0072	2.0073	2.0039	2.0130	2.0228	2.0482	2.1351	2.5120	3.3296	3.8402
KS-B3LYP	2.0530	2.0825	2.1394	2.2925	2.7973	3.6741	4.3134	—	—	—

On the other hand, the spin-restricted and spin-unrestricted energies for the lowest singlet state of *n*-coronene, obtained with the exact theory, should be the same, because of the symmetry constraint. To examine the possible symmetry-breaking effects, we additionally perform spin-restricted TAO-LDA, KS-LDA, and KS-B3LYP calculations for the lowest singlet energies on the respective optimized geometries. The spin-restricted and spin-unrestricted KS-LDA energies for the lowest singlet state of *n*-coronene are the same for the smaller *n*, but can be different for the larger *n*, due to the aforementioned spin contamination. Similar results are also found for KS-B3LYP. By contrast, the spin-restricted and spin-unrestricted TAO-LDA energies for the lowest singlet state of *n*-coronene are essentially the same (*i.e.*, within the numerical accuracy of our calculations), suggesting that essentially no unphysical symmetry-breaking effects occur in our spin-unrestricted TAO-LDA calculations. On the basis of our TAO-LDA results, *E*_ST_ monotonically decreases as *n* increases, and the ground states of *n*-coronenes are singlets for *n* = 2–11 (*i.e.*, all the cases studied).

### B. Vertical ionization potential, vertical electron affinity, and fundamental gap

At the ground-state (*i.e.*, the lowest singlet state) geometry of *n*-coronene (with *N* electrons), spin-unrestricted TAO-LDA is employed to calculate the vertical ionization potential1
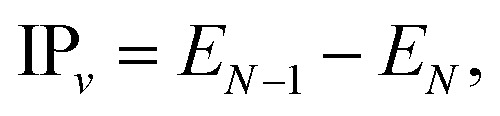
vertical electron affinity2
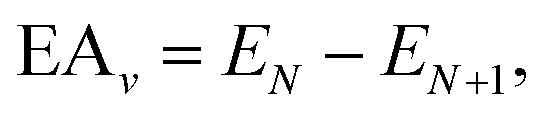
and fundamental gap3
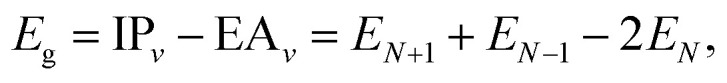
where *E*_*N*_ is the total energy of the *N*-electron system.

**Fig. 3:**
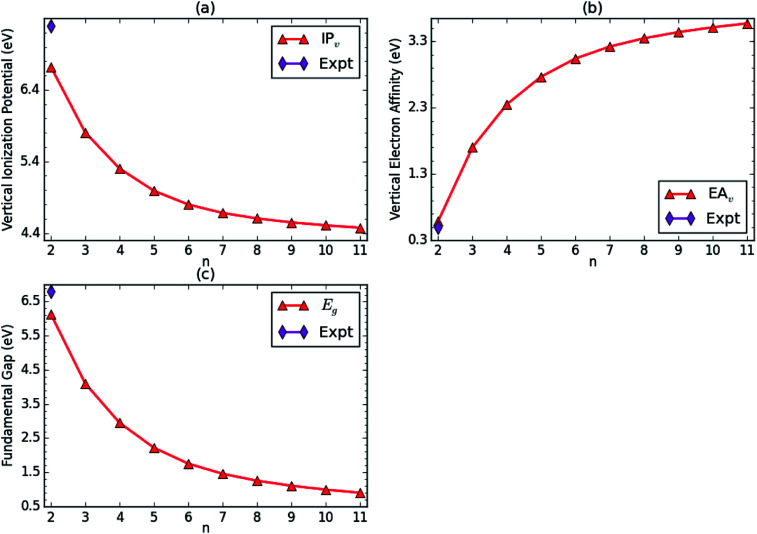
(a) Vertical ionization potential, (b) vertical electron affinity, and (c) fundamental gap for the lowest singlet state of *n*-coronene, obtained with spin-unrestricted TAO-LDA. Here, the experimental data are taken from the literature.^63–65^

With the increase of the side length of *n*-coronene, IP_*v*_ monotonically decreases, and EA_*v*_ monotonically increases, leading to a monotonically decreasing *E*_g_ (see Fig. 3). For *n* = 2, our TAO-LDA results are in reasonable agreement with the available experimental data.^63–65^ Note also that the calculated *E*_g_ value of *n*-coronene (*n* = 4–9) is within the most interesting range (1 to 3 eV), showing promise for applications of *n*-coronenes in nanophotonics (see Table S3 in ESI†).

### C. Symmetrized von Neumann entropy

In order to assess the possible polyradical character of *n*-coronene, we calculate the symmetrized von Neumann entropy (*e.g.*, see eqn (9) of ref. 37)4
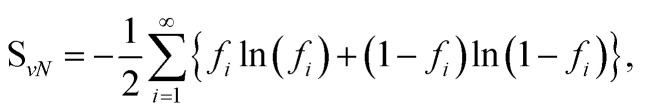
for the lowest singlet state of *n*-coronene, using spin-restricted TAO-LDA. In eqn (4), *f*_*i*_ (varying from 0 to 1) is the occupation number of the *i*th orbital obtained with TAO-LDA, which is qualitatively similar to the occupation number of the *i*th natural orbital.^29,35,38^ For a system without strong static correlation ({*f*_*i*_} are close to either 0 or 1), S_*vN*_ yields insignificant contributions, while for a system with strong static correlation ({*f*_*i*_} are fractional for active orbitals, and are close to either 0 or 1 for others), S_*vN*_ increases with the number of active orbitals.

**Fig. 4:**
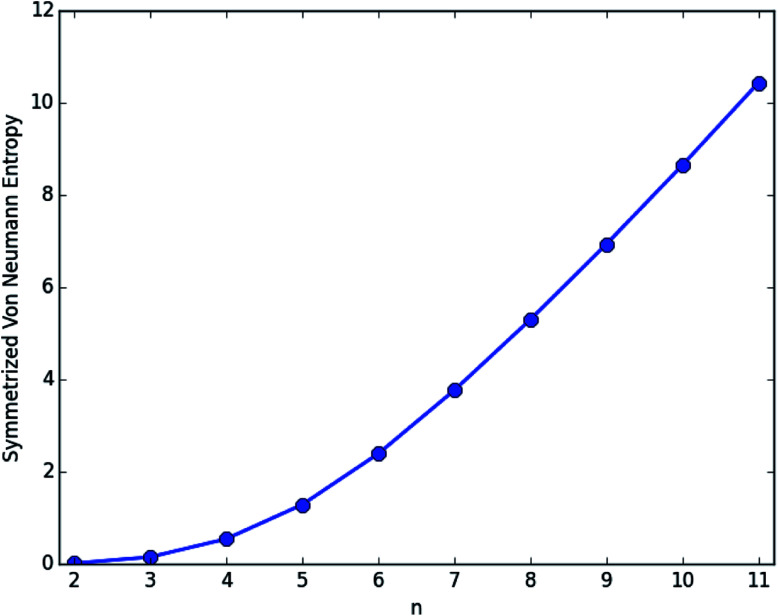
Symmetrized von Neumann entropy for the lowest singlet state of *n*-coronene, obtained with spin-restricted TAO-LDA.

As presented in Fig. 4, the S_*vN*_ value of *n*-coronene monotonically increases with increasing *n*, implying that the larger *n*-coronenes should possess increasing polyradical character in their ground states (see Table S4 in ESI†).

### D. Active orbital occupation numbers

To illustrate the reasons of the increase of S_*vN*_ with *n*, we plot the active orbital occupation numbers for the lowest singlet state of *n*-coronene, obtained with spin-restricted TAO-LDA. Here, the HOMO is the (*N*/2)th orbital, and the LUMO is the (*N*/2 + 1)th orbital, with *N* being the number of electrons in *n*-coronene. For brevity, HOMO, HOMO−1, …, and HOMO−8 are denoted as H, H−1, …, and H−8, respectively, while LUMO, LUMO+1, …, and LUMO+8 are denoted as L, L+1, …, and L+8, respectively.

**Fig. 5:**
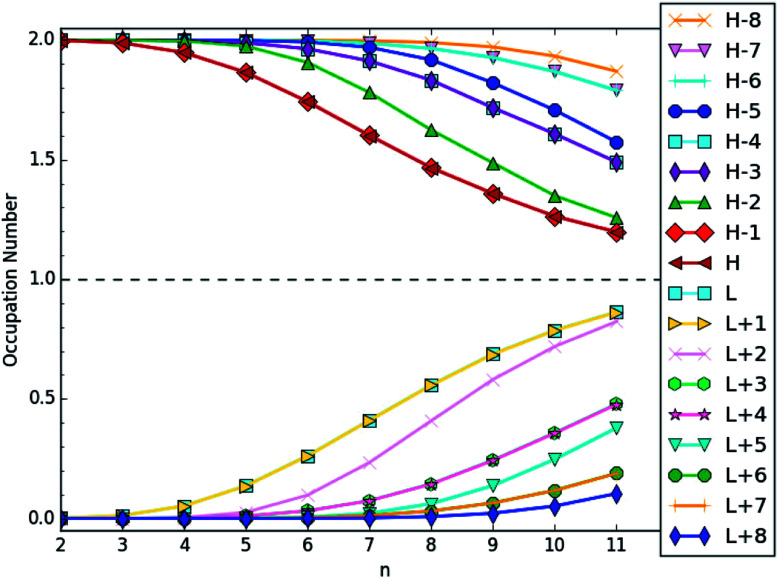
Active orbital occupation numbers (HOMO−8, …, HOMO−1, HOMO, LUMO, LUMO+1, …, and LUMO+8) for the lowest singlet state of *n*-coronene, obtained with spin-restricted TAO-LDA. For brevity, HOMO is denoted as H, LUMO is denoted as L, and so on.

As shown in Fig. 5, the number of fractionally occupied orbitals increases with increasing *n*, clearly demonstrating that the polyradical character of *n*-coronene indeed increases with the side length. On the basis of our TAO-LDA results, the smaller *n*-coronenes (*e.g.*, *n* < 5) possess non-radical character, and the larger *n*-coronenes (*e.g.*, *n* ≥ 5) possess increasing polyradical character in their ground states (see Table S5 in ESI†).

### E. Real-space representation of active orbitals

**Fig. 6:**
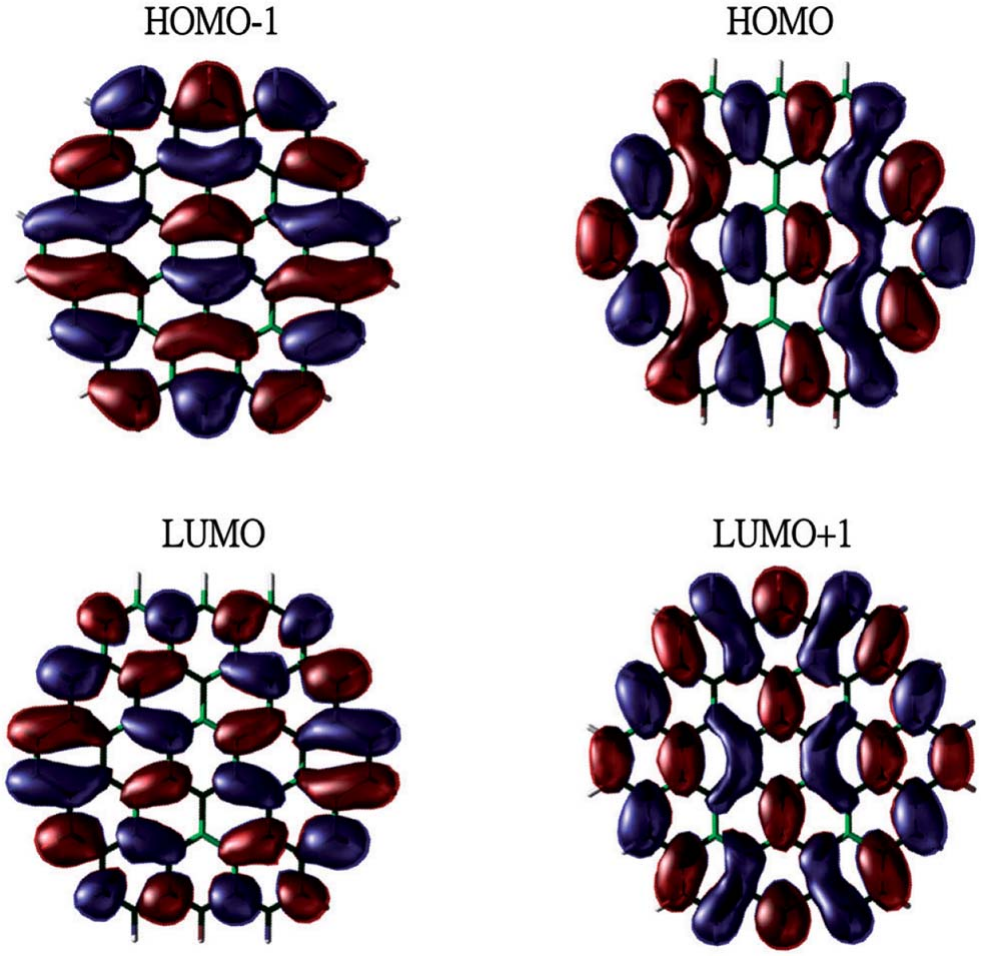
Real-space representation of the HOMO−1 (1.988), HOMO (1.988), LUMO (0.012), and LUMO+1 (0.012) for the lowest singlet state of 3-coronene, obtained with spin-restricted TAO-LDA, at isovalue = 0.01 e Å^−3^. The orbital occupation numbers are given in parentheses.

**Fig. 7:**
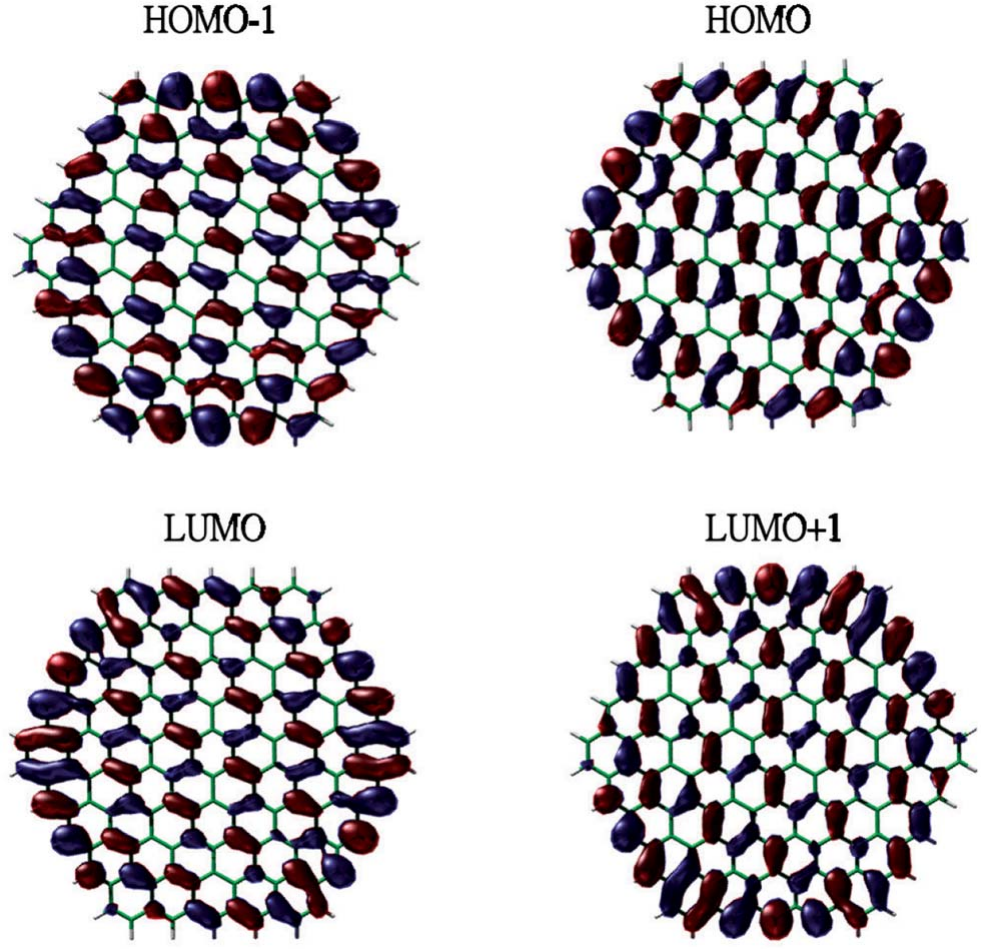
Real-space representation of the HOMO−1 (1.865), HOMO (1.864), LUMO (0.136), and LUMO+1 (0.136) for the lowest singlet state of 5-coronene, obtained with spin-restricted TAO-LDA, at isovalue = 0.01 e Å^−3^. The orbital occupation numbers are given in parentheses.

**Fig. 8:**
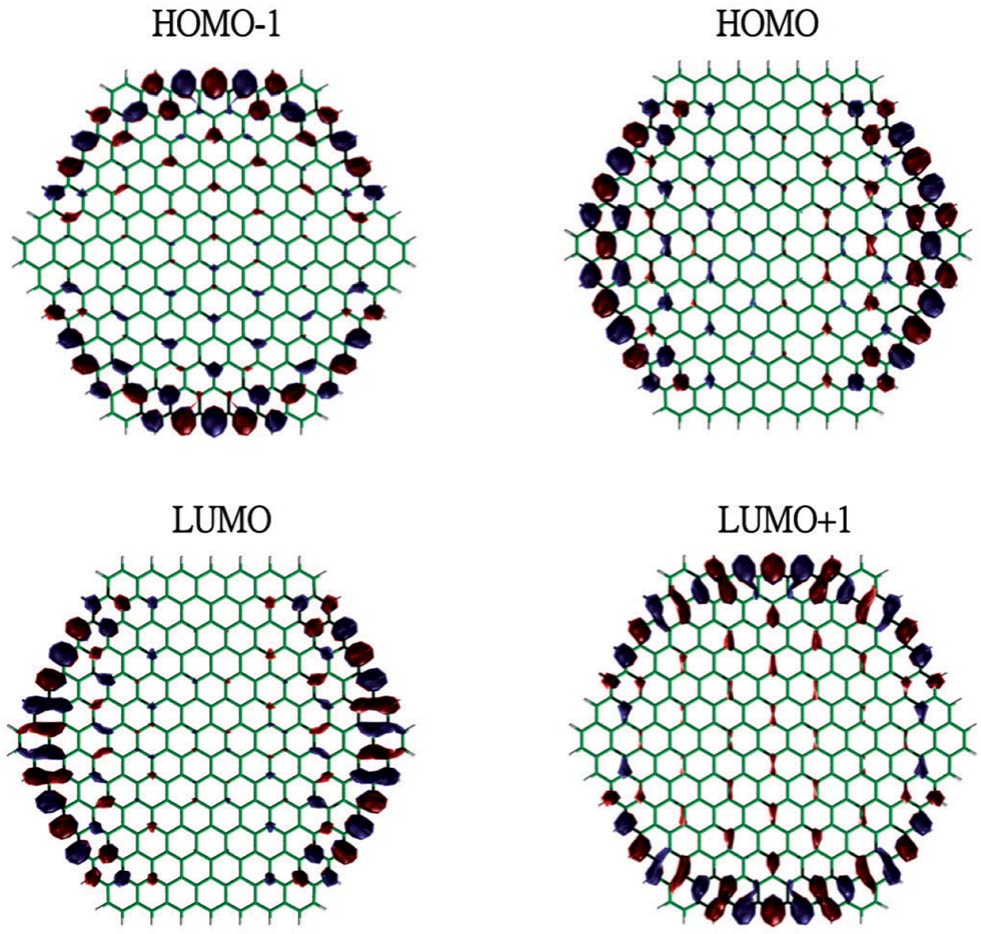
Real-space representation of the HOMO−1 (1.602), HOMO (1.601), LUMO (0.411), and LUMO+1 (0.409) for the lowest singlet state of 7-coronene, obtained with spin-restricted TAO-LDA, at isovalue = 0.01 e Å^−3^. The orbital occupation numbers are given in parentheses.

**Fig. 9:**
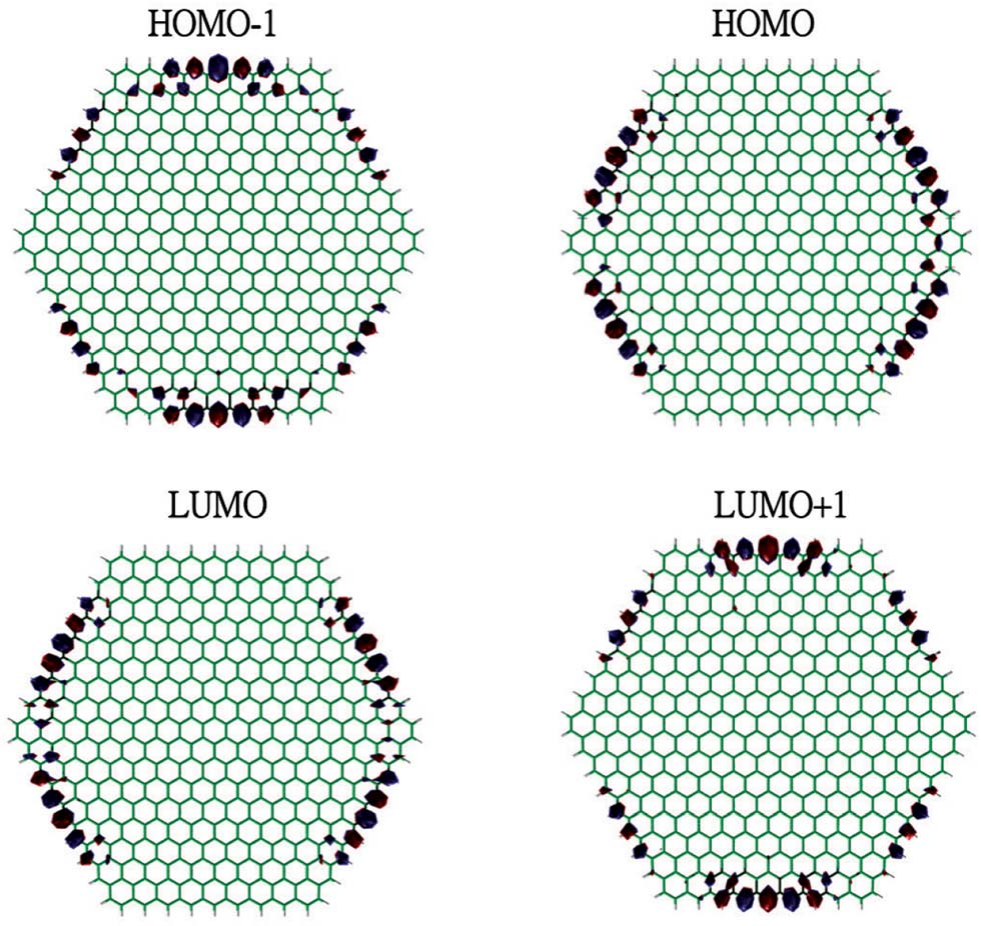
Real-space representation of the HOMO−1 (1.353), HOMO (1.352), LUMO (0.685), and LUMO+1 (0.684) for the lowest singlet state of 9-coronene, obtained with spin-restricted TAO-LDA, at isovalue = 0.01 e Å^−3^. The orbital occupation numbers are given in parentheses.

To show the transition from the non-radical character of the smaller *n*-coronenes to the increasing polyradical character of the larger *n*-coronenes in real space, we explore the real-space representation of active orbitals (*e.g.*, HOMO−1, HOMO, LUMO, and LUMO+1) for the lowest singlet states of some representative *n*-coronenes, obtained with spin-restricted TAO-LDA (see Fig. 6–9). For each *n*-coronene, the HOMO−1 and HOMO are nearly degenerate (and hence, complementary), and so are the LUMO and LUMO+1. For the smaller *n*-coronene (*e.g.*, *n* < 5), the active orbitals are delocalized over the whole molecule. However, with the increase of the side length of *n*-coronene, the active orbitals have an increased tendency to localize at the zigzag edges. For the larger *n*-coronene (*e.g.*, *n* ≥ 5), the active orbitals are mainly localized at the zigzag edges. Similar to previous findings for zigzag GNRs^32,36,39^ and cyclacenes,^55^ the increasing polyradical character of the larger *n*-coronenes should be closely correlated with the localization of active orbitals at the zigzag edges, which increases with the increase of the side length.

## IV. Conclusions

In conclusion, we have presented a systematic computational study on the electronic properties of *n*-coronenes (*n* = 2–11), involving *E*_ST_, IP_*v*_, EA_*v*_, *E*_g_, S_*vN*_, active orbital occupation numbers, and real-space representation of active orbitals, using the recently proposed TAO-DFT. While KS-DFT with the widely used XC density functionals is computationally efficient, it can yield enormous errors for systems possessing radical character. On the other hand, high-level *ab initio* multi-reference calculations are prohibitively expensive for large systems (especially for geometry optimization). Consequently, TAO-DFT is an ideal electronic structure method for the study of the larger *n*-coronenes (*e.g.*, *n* ≥ 5). The specific advances obtained with our TAO-DFT findings are as follows. First, our results are in reasonable agreement with the available experimental data. Secondly, from our TAO-DFT results, the ground states of *n*-coronenes are singlets for *n* = 2–11 (*i.e.*, all the cases studied). Thirdly, with the increase of the side length of *n*-coronene, *E*_ST_, IP_*v*_, and *E*_g_ decrease monotonically, while EA_*v*_ and S_*vN*_ increase monotonically. Finally, with increasing *n*, there is a transition from the non-radical character of the smaller *n*-coronenes (*e.g.*, *n* < 5) to the increasing polyradical character of the larger *n*-coronenes (*e.g.*, *n* ≥ 5). Moreover, the latter should be closely related to the localization of active orbitals at the zigzag edges, which increases with the increase of the side length.

Our work has further extended the understanding of correlations between the electronic properties and geometrical arrangements of 2D hydrocarbons. With the more 2D structures investigated, we believe that a solid foundation for a universal theory qualitatively describing the effect of the geometrical arrangements of 2D hydrocarbons on their electronic properties can be properly established. However, in view of the approximate nature of TAO-LDA, to examine the accuracy of our results, the electronic properties of *n*-coronenes from relatively affordable *ab initio* multi-reference methods are called for.

## Conflicts of interest

There are no conflicts to declare.

## Supplementary Material

RA-008-C8RA01336E-s001

## References

[cit1] Novoselov K. S. (2005). et al.. Nature.

[cit2] Zhang Y., Tan Y.-W., Stormer H. L., Kim P. (2005). Nature.

[cit3] Castro Neto A. H. (2009). et al.. Rev. Mod. Phys..

[cit4] Sun Z. (2010). et al.. Nature.

[cit5] Suenaga K., Koshino M. (2010). Nature.

[cit6] Wu Y. Q. (2011). et al.. Nature.

[cit7] Fujita M., Wakabayashi K., Nakada K., Kusakabe K. (1996). J. Phys. Soc. Jpn..

[cit8] Nakada K., Fujita M., Dresselhaus G., Dresselhaus M. S. (1996). Phys. Rev. B.

[cit9] Wakabayashi K., Fujita M., Ajiki H., Sigrist M. (1999). Phys. Rev. B.

[cit10] Liu R. (2011). et al.. J. Am. Chem. Soc..

[cit11] Lu J. (2011). et al.. Nat. Nanotechnol..

[cit12] Peng J. (2012). et al.. Nano Lett..

[cit13] Chua C. K. (2015). et al.. ACS Nano.

[cit14] Zen J. (2014). et al.. Astrophys. J., Lett..

[cit15] Vo T. H. (2014). et al.. Nat. Commun..

[cit16] Pan D. (2010). et al.. Adv. Mater..

[cit17] Shen J. (2011). et al.. Chem. Commun..

[cit18] Kim S. (2012). et al.. ACS Nano.

[cit19] Novoselov K. S. (2004). et al.. Science.

[cit20] Yang X. (2008). et al.. J. Am. Chem. Soc..

[cit21] Tapasztó L., Dobrik G., Lambin P., Biró L. (2008). Nat. Nanotechnol..

[cit22] Kosynkin D. V. (2009). et al.. Nature.

[cit23] Jiao L., Zhang L., Wang X., Diankov G., Dai H. (2009). Nature.

[cit24] Dutta S., Pati S. (2010). J. Mater. Chem..

[cit25] Clar E. , The Aromatic Sextet, John Wiley & Sons, New York, 1972

[cit26] Fujii S., Enoki T. (2013). Acc. Chem. Res..

[cit27] Sakamoto K., Nishina N., Enoki T., Aihara J.-i. (2014). J. Phys. Chem. A.

[cit28] Trinquier G., Malrieu J.-P. (2015). Chem. - Eur. J..

[cit29] Yeh C.-N., Chai J.-D. (2016). Sci. Rep..

[cit30] Kivelson S., Chapman O. L. (1983). Phys. Rev. B.

[cit31] Bendikov M. (2004). et al.. J. Am. Chem. Soc..

[cit32] Hachmann J., Dorando J. J., Aviles M., Chan G. K. L. (2007). J.
Chem. Phys..

[cit33] Hajgató B., Szieberth D., Geerlings P., De Proft F., Deleuze M. S. (2009). J. Chem. Phys..

[cit34] Huzak M., Deleuze M. S., Hajgató B. (2011). J. Chem. Phys..

[cit35] Chai J.-D. (2012). J. Chem. Phys..

[cit36] Mizukami W., Kurashige Y., Yanai T. (2013). J. Chem. Theory Comput..

[cit37] Rivero P., Jiménez-Hoyos C. A., Scuseria G. E. (2013). J. Phys. Chem. B.

[cit38] Chai J.-D. (2014). J. Chem. Phys..

[cit39] Wu C.-S., Chai J.-D. (2015). J. Chem. Theory Comput..

[cit40] Fosso-Tande J., Nguyen T.-S., Gidofalvi G., DePrince III A. E. (2016). J. Chem. Theory Comput..

[cit41] Seenithurai S., Chai J.-D. (2016). Sci. Rep..

[cit42] Chai J.-D. (2017). J. Chem. Phys..

[cit43] Ikäläinen S. (2009). et al.. Phys. Chem. Chem. Phys..

[cit44] Philpott M. R., Kawazoe Y. (2009). J. Chem. Phys..

[cit45] Jochims H. W. (1994). et al.. Astrophys. J..

[cit46] Le Page V. (1997). et al.. J. Am. Chem. Soc..

[cit47] Schröder D. (2001). et al.. Helv. Chim. Acta.

[cit48] Koskinen P., Malola S., Häkkinen H. (2008). Phys. Rev. Lett..

[cit49] Herbst E., Van Dishoeck E. F. (2009). Annu. Rev. Astron. Astrophys..

[cit50] Abouaf R., Diaz-Tenderoa S. (2009). Phys. Chem. Chem. Phys..

[cit51] Kohn W., Sham L. J. (1965). Phys. Rev..

[cit52] Cohen A. J., Mori-Sánchez P., Yang W. (2012). Chem. Rev..

[cit53] Gryn’ova G., Coote M. L., Corminboeuf C. (2015). Wiley Interdiscip. Rev.: Comput. Mol. Sci..

[cit54] Lin C.-Y., Hui K., Chung J.-H., Chai J.-D. (2017). RSC Adv..

[cit55] Wu C.-S., Lee P.-Y., Chai J.-D. (2016). Sci. Rep..

[cit56] Seenithurai S., Chai J.-D. (2017). Sci. Rep..

[cit57] Löwdin P.-O., Shull H. (1956). Phys. Rev..

[cit58] Shao Y. (2015). et al.. Mol. Phys..

[cit59] Dirac P. A. M. (1930). Proc. Cambridge Philos. Soc..

[cit60] Perdew J. P., Wang Y. (1992). Phys. Rev. B.

[cit61] Stephens P. J., Devlin F. J., Chabalowski C. F., Frisch M. J. (1994). J. Phys. Chem..

[cit62] Young D. C. , Computational Chemistry: A Practical Guide for Applying Techniques to Real World Problems, Wiley, New York, 2001

[cit63] Malloci G., Joblin C., Mulas G. (2007). Astron. Astrophys..

[cit64] Duncan M. A. (1999). et al.. Chem. Phys. Lett..

[cit65] Chen G., Cookscor R. G., Corpuz E., Scott L. T. (1996). J. Am. Soc. Mass Spectrom..

