# Supplementary material for: Electronic properties of the coronene series from thermally-assisted-occupation density functional theory
Source: RSC Adv. 2018 Oct 8;8(60):34350–8. doi: 10.1039/c8ra01336e (PMC9087050; doi:10.1039/c8ra01336e)
Supplement: RA-008-C8RA01336E-s001 [file RA-008-C8RA01336E-s001.pdf]

Electronic Supplementary Material (ESI) for RSC Advances.
This journal is © The Royal Society of Chemistry 2018

Supplementary Material to: Electronic Properties of the Coronene Series from Thermally-Assisted-Occupation Density Functional Theory

Chia-Nan Yeh,[1] Can Wu,[2] Haibin Su,[2,3,*] and Jeng-Da Chai[1,4,5,†]

[1]*Department of Physics, National Taiwan University, Taipei 10617, Taiwan*

[2]*School of Materials Science and Engineering, Nanyang Technological University, 50 Nanyang Avenue, Singapore 639798, Republic of Singapore*

[3]*Department of Chemistry, The Hong Kong University of Science and Technology, Hong Kong, China*

[4]*Center for Theoretical Physics, National Taiwan University, Taipei 10617, Taiwan*

[5]*Center for Quantum Science and Engineering, National Taiwan University, Taipei 10617, Taiwan*

[*] Corresponding author. Electronic mail: haibinsu@ust.hk

[†] Corresponding author. Electronic mail: jdchai@phys.ntu.edu.tw

LIST OF TABLES

S1 Comparison of the singlet-triplet energy gap E_{ST} (in kcal/mol) of n-coronene, calculated using spin-unrestricted TAO-LDA with the 6-31G(d) and 6-31G basis sets. 3

S2 Singlet-triplet energy gap E_{ST} (in kcal/mol) of n-coronene, calculated using spin-unrestricted TAO-LDA, KS-LDA, and KS-B3LYP with the 6-31G basis set. Here, the experimental data is taken from the literature [1]. 4

S3 Vertical ionization potential IP_v (in eV), vertical electron affinity EA_v (in eV), and fundamental gap E_g (in eV) for the lowest singlet state of n-coronene, calculated using spin-unrestricted TAO-LDA with the 6-31G basis set. Here, the experimental data (given in parentheses) are taken from the literature [2–4]. 5

S4 Symmetrized von Neumann entropy S_{vN} for the lowest singlet state of n-coronene, calculated using spin-restricted TAO-LDA with the 6-31G basis set. 6

S5 Active orbital occupation numbers (HOMO−8, ..., HOMO−1, HOMO, LUMO, LUMO+1, ..., and LUMO+8) for the lowest singlet state of n-coronene, calculated using spin-restricted TAO-LDA with the 6-31G basis set. For brevity, HOMO is denoted as H, LUMO is denoted as L, and so on. . 7

TABLE S1. Comparison of the singlet-triplet energy gap E_{ST} (in kcal/mol) of n-coronene, calculated using spin-unrestricted TAO-LDA with the 6-31G(d) and 6-31G basis sets.

n	6-31G(d)	6-31G	Difference
2	51.31	52.41	-1.10
3	30.05	30.81	-0.76
4	17.68	18.16	-0.48
5	9.96	10.21	-0.25
6	5.49	5.59	-0.10
7	3.18	3.21	-0.03
8	2.02	2.03	-0.01
9	1.41	1.40	0.01

TABLE S2. Singlet-triplet energy gap E_{ST} (in kcal/mol) of n-coronene, calculated using spin-unrestricted TAO-LDA, KS-LDA, and KS-B3LYP with the 6-31G basis set. Here, the experimental data is taken from the literature [1].

n	TAO-LDA	KS-LDA	KS-B3LYP	Expt.
2	52.41	60.21	61.10	55.35 [1]
3	30.81	40.77	43.23	
4	18.16	28.94	31.35	
5	10.21	20.82	22.05	
6	5.59	14.86	13.31	
7	3.21	10.31	2.91	
8	2.03	6.78	-9.06	
9	1.40	3.77		
10	1.05	0.53		
11	0.82	1.34		

TABLE S3. Vertical ionization potential IP_v (in eV), vertical electron affinity EA_v (in eV), and fundamental gap E_g (in eV) for the lowest singlet state of n-coronene, calculated using spin-unrestricted TAO-LDA with the 6-31G basis set. Here, the experimental data (given in parentheses) are taken from the literature [2–4].

n	IP_v	EA_v	E_g
2	6.72 (7.29 [2])	0.58 (0.50 [3, 4])	6.14 (6.79 [2–4])
3	5.81	1.70	4.11
4	5.30	2.35	2.95
5	4.99	2.77	2.22
6	4.80	3.04	1.76
7	4.68	3.22	1.46
8	4.60	3.35	1.26
9	4.55	3.44	1.11
10	4.51	3.51	0.99
11	4.47	3.57	0.90

TABLE S4. Symmetrized von Neumann entropy S_{vN} for the lowest singlet state of n-coronene, calculated using spin-restricted TAO-LDA with the 6-31G basis set.

n	S_{vN}
2	0.01
3	0.15
4	0.54
5	1.29
6	2.39
7	3.77
8	5.30
9	6.93
10	8.65
11	10.44

TABLE S5. Active orbital occupation numbers (HOMO−8, ..., HOMO−1, HOMO, LUMO, LUMO+1, ..., and LUMO+8) for the lowest singlet state of n-coronene, calculated using spin-restricted TAO-LDA with the 6-31G basis set. For brevity, HOMO is denoted as H, LUMO is denoted as L, and so on.

n	2	3	4	5	6	7	8	9	10	11
H−8	2.000	2.000	2.000	2.000	2.000	1.997	1.990	1.971	1.933	1.870
H−7	2.000	2.000	2.000	1.999	1.996	1.987	1.965	1.927	1.869	1.791
H−6	2.000	2.000	2.000	1.999	1.996	1.987	1.965	1.927	1.868	1.790
H−5	2.000	2.000	2.000	1.999	1.992	1.970	1.917	1.828	1.707	1.574
H−4	2.000	2.000	1.998	1.989	1.964	1.913	1.833	1.728	1.609	1.491
H−3	2.000	2.000	1.998	1.989	1.964	1.913	1.832	1.727	1.608	1.490
H−2	2.000	2.000	1.997	1.975	1.907	1.781	1.624	1.474	1.350	1.257
H−1	1.999	1.988	1.948	1.865	1.743	1.602	1.467	1.353	1.264	1.198
H	1.999	1.988	1.948	1.864	1.742	1.601	1.466	1.352	1.263	1.197
L	0.001	0.012	0.052	0.136	0.262	0.411	0.557	0.685	0.787	0.863
L+1	0.001	0.012	0.052	0.136	0.261	0.409	0.556	0.684	0.786	0.862
L+2	0.000	0.000	0.003	0.025	0.097	0.234	0.408	0.578	0.719	0.823
L+3	0.000	0.000	0.002	0.010	0.032	0.074	0.144	0.241	0.357	0.478
L+4	0.000	0.000	0.002	0.010	0.031	0.074	0.144	0.240	0.356	0.477
L+5	0.000	0.000	0.000	0.001	0.005	0.021	0.061	0.136	0.247	0.378
L+6	0.000	0.000	0.000	0.001	0.004	0.012	0.031	0.065	0.117	0.189
L+7	0.000	0.000	0.000	0.001	0.004	0.012	0.031	0.064	0.117	0.188
L+8	0.000	0.000	0.000	0.000	0.001	0.002	0.007	0.022	0.052	0.104

[1] R. Abouaf and S. Diaz-Tenderoa, *Phys. Chem. Chem. Phys.*, 2009, **11**, 5686–5694.

[2] G. Malloci, C. Joblin, and G. Mulas, *Astron. Astrophys.*, 2007, **462**, 627–635.

[3] M. A. Duncan *et al.*, *Chem. Phys. Lett.*, 1999, **309**, 49–54.

[4] G. Chen, R. G. Cookscor, E. Corpuz, and L. T. Scott, *J. Am. Soc. Mass Spectrosc.*, 1996, **7**, 619–627.
